# Biological sex, but not obesity, independently predicts anatomical injury patterns in trauma bay patients: a multivariate retrospective analysis of 2,128 patients

**DOI:** 10.1186/s13293-026-00883-z

**Published:** 2026-03-21

**Authors:** C. Pietsch, B. Erdle, F. Klingler, F. C. Wagner, J. P. Maier, H. Schmal, N. Mühlenfeld

**Affiliations:** 1 Department of Orthopedics and Trauma Surgery, Faculty of Medicine, Medical Centre- Albert-Ludwig’s-University of Freiburg, Albert- Ludwig’s-University of Freiburg, Hugstetter Str. 55, 79106 Freiburg, Germany; 2 Department of Orthopedic Surgery, University Hospital Odense, Sdr. Boulevard 29, Odense C, 5000 Denmark

**Keywords:** Obesity, Injury Severity, Anthropometric sex differences, Sex-specific bone mineral density, Hormonal influence on tissue elasticity

## Abstract

**Background:**

While sex-specific disparities in trauma outcomes are well established; the biological contribution of sex versus anthropometric variables like obesity in determining initial anatomical injury severity remains poorly understood. This study aimed to independently isolate the effect of biological sex on serious damage, while controlling for energy exposure and body factors.

**Methods:**

A retrospective cohort study of 2,128 consecutive adult trauma bay patients (males: *n* = 1570; females: *n* = 558) was conducted. Univariate comparisons and multivariate binary logistic regression models were used to identify independent predictors of severe overall injury (Injury Severity Score [ISS] ≥ 9) and severe regional injuries (Abbreviated Injury Scale [AIS] ≥ 3), while controlling for age, body-mass-index (BMI) and obesity (BMI ≥ 30). Adjusted odds ratios (aOR) were calculated with 95% confidence intervals.

**Results:**

Compared to females, males were significantly more likely to suffer High-Energy Trauma (HET) (*p* < 0.001) and had a higher overall injury severity (ISS ≥ 9) in univariate analysis. Male sex was found to be an independent predictor of significant injury in all adjusted models: ISS ≥ 9 (aOR = 1.33, *p* = 0.010), AIS ≥ 3 for thorax/spine (aOR = 1.38, *p* = 0.003), and AIS ≥ 3 for face (aOR = 1.78, *p* = 0.020). For ISS ≥ 9 (aOR = 1.27, *p* = 0.034) and AIS ≥ 3 thorax/spine (aOR = 2.40, *p* < 0.001), HET was an independent predictor. Neither dichotomous obesity (BMI ≥ 30) nor continuous BMI was a significant independent predictor of any injury outcome (all *p* ≥ 0.075), with odds ratios near unity (OR range: 0.978–1.017) indicating no dose-response relationship.

**Conclusions:**

Biological sex and kinetic energy are the primary determinants for severe overall and regional trauma. Conversely, obesity does not independently affect initial anatomical injury severity. The lack of independent impact from obesity suggests that sexual dimorphism in biomechanics, skeletal geometry, and tissue resilience – rather than absolute obesity – underlies the increased vulnerability of males in acute trauma.

## Abstract (plain language summary)

This study analyzed 2,128 trauma patients to determine how biological sex, age, body mass index (BMI) and obesity (BMI ≥ 30) influence the severity of initial injuries.

We found that male sex is a significant, independent predictor of severe injury. Even when accounting for the fact that men are more likely to be involved in high-energy accidents, they still sustain more severe injuries to the chest, spine, and face than females in similar situations. Contrary to some theories that body fat might provide “padding” or, conversely, increase injury risk, this study found that obesity does not independently affect the severity of initial injury. While obesity may cause complications later during recovery, it doesn’t change how badly the body is physically damaged at the moment of impact. As expected, high-energy trauma (HET) is the strongest predictor of severe chest and spinal injuries. Our findings suggest that men’s higher injury rates aren’t just about “risky behavior”. Instead, they point to biological factors: differences in bone density and face shape (like a more protruding jaw or brow) could lead to more severe fractures, variations in hormones and muscle distribution could change how the body absorbs force and, most vehicle safety systems (like seatbelts) have historically been designed using male-based data, which may affect how energy is transferred to the body.

Clinicians should view male sex as a high-risk factor for severe internal and facial damage independent of a patient’s BMI. This supports the use of more aggressive early imaging for male trauma patients.

## Highlights

This study of 2,128 patients identifies biological sex and kinetic energy - not obesity - as the primary independent drivers of initial trauma severity

Male sex is an independent predictor of severe injury (Injury Severity Score [ISS] ≥ 9). Even when adjusting for energy exposure, men have a 78% higher risk of severe facial injuries and a 38% higher risk of chest/spine injuries compared to the female population

Obesity does not impact initial injury severity independently. The "cushioning effect" does not apply in acute trauma; obesity's known risks likely result from later complications rather than the impact itself

Differences in bone geometry, muscle tension ("bracing"), and tissue elasticity result in male bodies absorbing kinetic energy differently, leading to higher injury burden

Triage protocols should prioritize aggressive diagnostics in male patients, regardless of body habitus

## Background

Trauma remains among the leading causes of injury and death globally [1–3], calling for constant enhancement of triage and treatment procedures. Significant differences in injury severity, causes, and clinical outcomes continue to exist between demographic groups, particularly between male and female patients [4–6], despite standardized protocols like Advanced Trauma Life Support (ATLS) aiming for consistent therapy.

While prior research frequently attributed disparities in overall survival to factors like age and pre-existing comorbidities [7, 8], the specific function of sex as an independent predictor of injury severity—especially after correcting for confounding factors—remains incompletely understood. Controlling for significant confounding variables, especially the mechanism of injury and body habitus, is a key issue in determining the true effect of sex on injury severity [9]. Males are epidemiologically known to be more commonly exposed to high-velocity, HET mechanisms [10–12], which intrinsically result in increased injury severity. Additionally, there is conflicting evidence in the literature regarding the effect of obesity on the severity of initial injuries, while age is a well-known strong prognostic factor [13–15]. While some studies suggest that adipose tissue provides a protective “padding” effect [15, 16], others link obesity to higher comorbidity rates and worse outcomes [17]. Disentangling the independent contribution of sex from these significant modifiers is critical for appropriate risk categorization.

The physiological and biomechanical response to traumatic force is fundamentally modulated by biological sex [17]. Beyond differing risk-taking behaviors, sexual dimorphism in skeletal density, ligamentous laxity, and fat distribution patterns may alter how kinetic energy is dissipated through the body. While previous literature often focuses on the ‘obesity paradox’ in long-term recovery [15], the independent role of biological sex in the hyperacute phase - detached from the confounding effects of obesity and injury mechanism - remains a critical gap in trauma research. Understanding whether male vulnerability is a result of higher energy exposure or intrinsic biological susceptibility is essential for sex-aware emergency diagnostics.

Therefore, this study attempted to determine the sex-specific differences in trauma mechanism and injury severity in a large cohort of consecutively hospitalized trauma bay patients of a level I trauma center. We specifically hypothesized that:

1. Epidemiological Sex Disparity: Males would exhibit a significantly higher prevalence of HET mechanisms in comparison to females – reflecting sex-specific exposure patterns.
2. Biological Vulnerability: Biological male sex is an independent risk factor for severe injury (Injury Severity Score [ISS] ≥ 9), particularly for specific high-impact regional injuries including Abbreviated Injury Scale (AIS) ≥ 3 injuries to the chest/thoracic spine and face, even after controlling for major confounders such as age and obesity – suggesting intrinsic physiological or biomechanical susceptibility.

This study attempts to clarify the independent impact of sex on the initial traumatic burden and contributes to a more complex, sex-aware approach to trauma risk assessment by employing multivariate analysis to properly adjust for these important variables.

## Methods

This study was a retrospective, single-center cohort analysis performed at a certified Level I Trauma Center. This study followed the STROBE guidelines for observational studies (Strengthening the Reporting of Observational Studies in Epidemiology) and the RECORD guidelines (Reporting of studies Conducted using Observational Routinely collected Data) [18, 19].

The study cohort consisted of a consecutive series of all adult trauma patients (≥ 18 years) who were primarily admitted to the emergency trauma unit because of acute traumatic injuries between January 2018 and December 2024. Patients were included irrespective of injury severity or mechanism, thereby ensuring substantial clinical relevance and generalizability for a diverse trauma population. Patients transferred from other hospitals or those with isolated minor injuries that did not necessitate trauma team activation were excluded. The final cohort comprised 2128 patients.

Data were obtained from electronic patient records and the institutional trauma registry. The subsequent variables were gathered: Demographics and Risk Factors: Age (continuous, years), Sex (male/female), Body Mass Index (BMI, continuous, kg/m²), Smoking status, and Alcohol consumption (Yes/No). Injury Severity: The Injury Severity Score (ISS) and Abbreviated Injury Scale (AIS) were calculated and validated for all six anatomical regions: Head/Neck, Face, Chest/Thoracic Spine, Abdomen/Pelvis, Extremities/Pelvic Girdle, and External. Mechanism of Injury: Classified into High-Energy Trauma and Low-Energy Trauma according to the computed totals from the supplied tables. High-Energy Trauma (HET) was defined according to established trauma triage criteria and included: (1) motor vehicle crashes with speed > 60 km/h, intrusion > 30 cm, or ejection; (2) motorcycle crashes at any speed; (3) pedestrian or bicyclist struck by motor vehicle; (4) falls from height > 3 m; and (5) crush injuries. HET classification was determined by the attending trauma surgeon through trauma registry data and medical record documentation retrospectively. Low-Energy Trauma (LET) was defined as ground-level falls, low-speed motor vehicle crashes, and isolated penetrating injuries not meeting HET criteria.

Two principal binary outcomes, indicating severe overall injury and severe regional injury, were established:

1. Primary Outcome 1 (Overall Injury Severity): ISS ≥ 9 (Affirmative/Negative).
2. Primary Outcome 2 (Regional Injury Severity): Severe regional injury, characterized by an Abbreviated Injury Scale (AIS) ≥ 3 for the two anatomical regions, with significant sex-disparities in injury severity: the chest/thoracic spine and the face.

### Statistical analysis

All statistical analysis was performed using Graphpad Prism 10.6 (Graphpad, CA, San Diego). Continuous variables were expressed as median and interquartile range (IQR) because of non-normal distribution and were compared utilizing the Mann-Whitney U Test. Categorical variables were presented as frequency and percentage (n, %) and analyzed using Fisher’s Exact Test or the Chi-Square test, as applicable. A p-value of less than 0.05 was valued statistically significant. Separate multivariate binary logistic regression models were developed to ascertain the independent effect of sex on the two primary outcomes. The model predictors (independent variables) included sex (male vs. female, with female as the reference group), age (continuous, measured per year), and obesity (BMI ≥ 30, binary yes vs. no). In addition to the binary obesity variable (BMI ≥ 30), BMI was also entered as a continuous variable (per kg/m²) in separate multivariate models to evaluate potential dose–response relationships across the full BMI spectrum.

The initial models incorporating smoking and alcohol consumption were rendered mathematically unfeasible due to perfect separation, leading to the exclusion of these variables from the final adjusted models. The logistic regression results are displayed as Adjusted Odds Ratios (aOR) along with their respective 95% Confidence Intervals (CI) and p-values. The aOR for sex were inverted to reflect the risk for the male cohort (male vs. female reference) for enhanced clarity and clinical interpretability. A post hoc power analysis was conducted for the multivariate logistic regression models using the observed regression coefficient and standard error for the sex variable. Achieved power was calculated using a two-sided α of 0.05. Additionally, the minimum detectable odds ratio at 80% power was estimated for each endpoint.

In accordance with the Sex and Gender Equity in Research (SAGER) guidelines and the Sex as a Biological Variable (SABV) principle, biological sex was treated as a primary independent variable. Sex-disaggregated data was assessed to identify potential dimorphisms in injury patterns. Multivariate models were specifically designed to isolate the biological influence of biological sex by adjusting for external modifiers such as injury energy and body habitus, thereby allowing for an analysis of intrinsic sex-specific vulnerability.

## Results

**Fig. 1:**
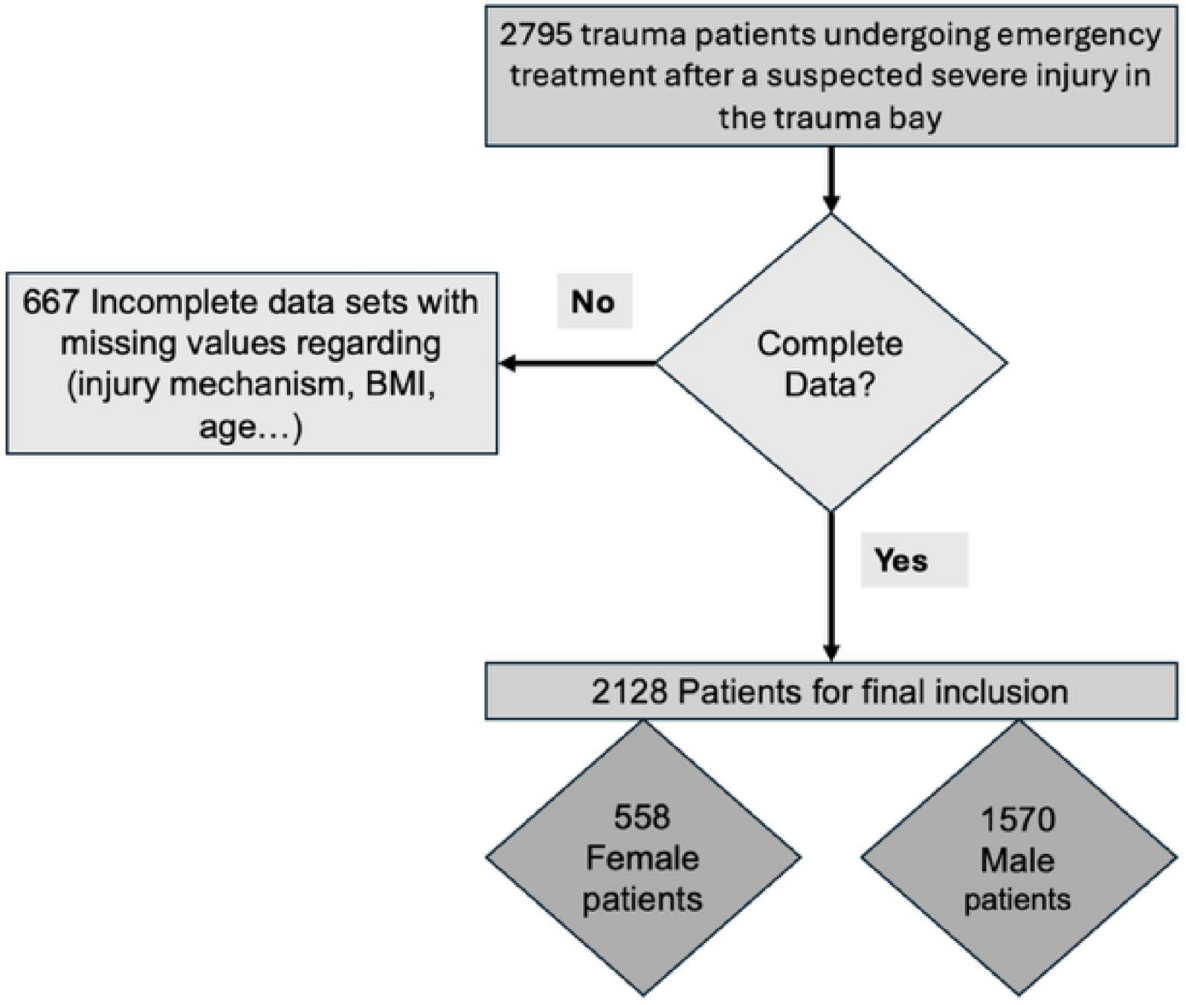
Patient flow chart illustrating flow for inclusion in our final data set of 2128 adult trauma patients

A total of 2128 trauma cases were analyzed, consisting of 1570 men (73.8%) and 558 females (26.2%) (Fig. 1.).

Significant disparities were noted between the sexes concerning demographics and injury prevalence (Table 1).

**Table 1:** This table compares the characteristics and injury severity between the female and male cohorts in our entire Trauma Bay sample (*n* = 2128)

Characteristic	Females (*n* = 558)	Males (*n* = 1570)	*p*-Value
**Demographics**			
Age (Median, IQR)	54.0 (30.0–73.0)	51.0 (32.0–63.0)	0.003*
**Comorbidities / Risk Factors**			
BMI ≥ 30 (n, %)	86 (15.4%)	245 (15.6%)	0.9291
BMI (Median, IQR)	24.2 (22.0-27.6)	25.5 (23.7–28.3)	< 0.001*
Smoking (Yes, n, %)	35 (6.3%)	123 (7.8%)	0.002*
Alcohol Consumption (Yes, n, %)	28 (5.0%)	110 (7.0%)	< 0.001*
**Injury Severity (Overall & Regional)**			
ISS (Median, IQR)	12.0 (5.0–22.0)	13.0 (6.0–22.0)	0.044*
ISS ≥ 9 (n, %)	353 (63.8%)	1090 (69.1%)	0.023*
AIS Head/Neck (Median, IQR)	2.0 (1.0–3.0)	2.0 (1.0–3.0)	0.845
AIS Face (Median, IQR)	1.0 (1.0–2.0)	1.0 (1.0–2.0)	0.003*
AIS Chest/thoracic spine (Median, IQR)	2.0 (2.0–3.0)	3.0 (2.0–3.0)	0.004*
AIS Abdomen/Pelvis (Median, IQR)	2.0 (2.0–3.0)	2.0 (2.0–3.0)	0.882
AIS Extremities/Pelvic Girdle (Median, IQR)	2.0 (1.0–3.0)	2.0 (1.0–3.0)	0.112
AIS External (Median, IQR)	1.0 (1.0–1.0)	1.0 (1.0–1.0)	0.343
AIS Thorax/Spine ≥ 3 (n, %)	156 (28.2%)	562 (35.8%)	0.001*
AIS Face ≥ 3 (n, %)	18 (3.2%)	91 (5.8%)	0.019*
**Mortality (n**,** %)**	33 (5.9%)	81 (5.2%)	0.506

*Statistically significant, IQR: interquartile range; BMI = body mass index; ISS = Injury Severity Score; AIS = Abbreviated Injury Scale

### Demographics and risk factors

Female patients were considerably older than male patients (median age: 54.0 years vs. 51.0 years, *p* = 0.003). The median BMI exhibited a significant difference, with females at 24.2 kg/m² and males at 25.5 kg/m² (*p* < 0.001). The percentage of patients classified as obese (BMI ≥ 30) was approximately the same and not statistically significant (15.4% for females compared to 15.6% for males, *p* = 0.929). Moreover, established risk factors such as smoking (6.3% vs. 7.8%, *p* = 0.002) and consumption of alcohol (5.0% vs. 7.0%, *p* < 0.001) were considerably more prevalent in the male cohort than in the female cohort.

### Injury severity (univariate)

The median ISS was marginally but significantly elevated for males, measuring 13.0 compared to 12.0 (*p* = 0.044). The percentage of patients with severe injury (ISS ≥ 9) was notably greater in males (69.1% vs. 63.8%, *p* = 0.023).

### Regarding regional injury severity (AIS)

Males demonstrated a markedly elevated median AIS compared to females for the chest/thoracic spine (3.0 vs. 2.0, *p* = 0.004) and face (1.0 vs. 1.0, *p* = 0.003). The percentage of patients with a serious thorax/spine injury (AIS ≥ 3) was markedly greater in males than in females (35.8% vs. 28.2%, *p* = 0.001). In addition, the incidence of serious facial injury (AIS ≥ 3) was significantly higher in males compared to females (91 patients, 5.8% vs. 18 patients, 3.2%, *p* = 0.019). The overall mortality rates were similar between the groups (5.9% vs. 5.2%, *p* = 0.506).

### Mechanism of Injury

**Fig. 2:**
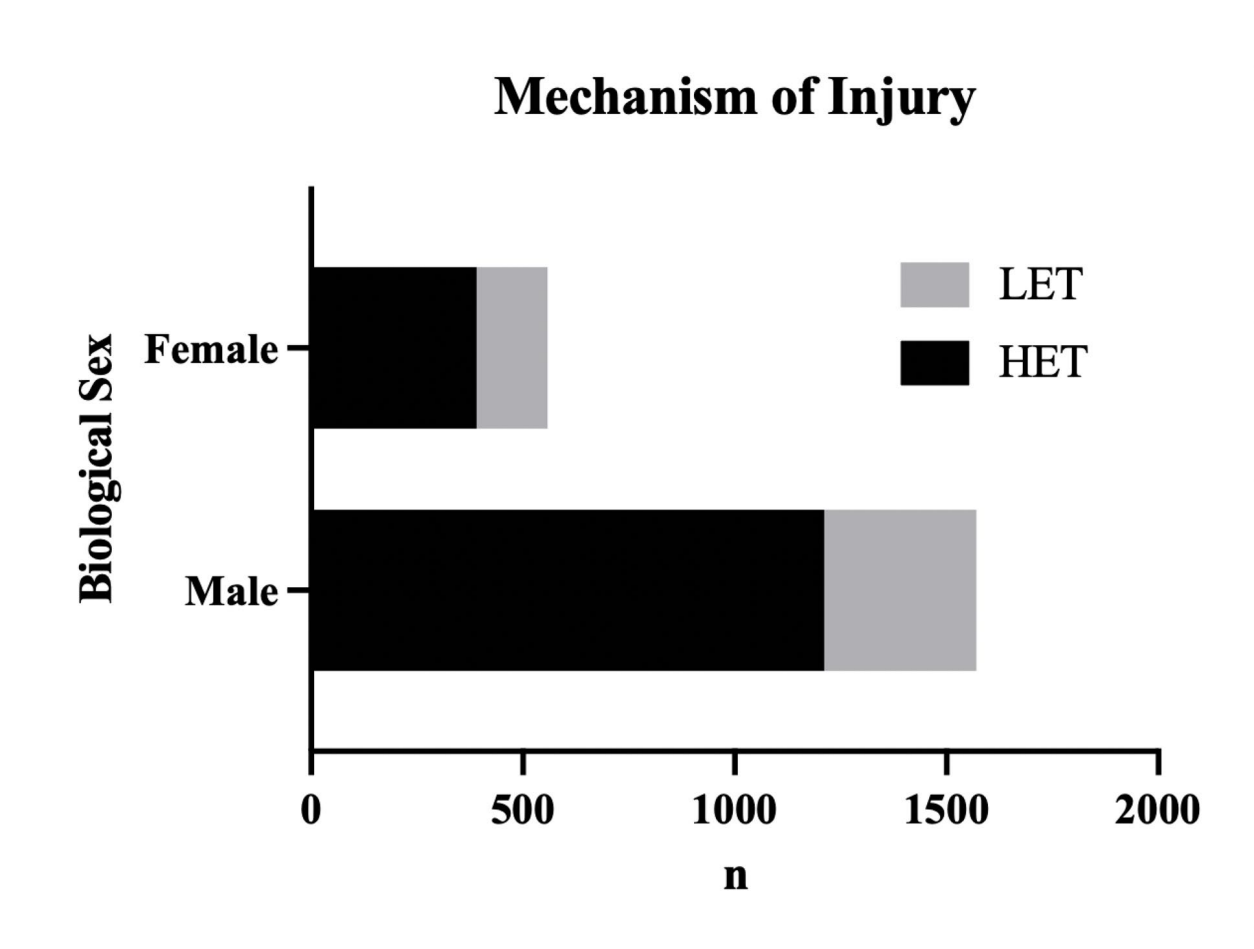
Mechanism of injury stratified by biological sex. The distribution of Low-Energy Trauma (LET, gray bars) and High-Energy Trauma (HET, black bars) is presented for female and male patients. Males demonstrated a higher frequency of both injury mechanisms compared to females, with HET being predominant in the male cohort

The distribution of injury mechanism varied significantly between sex (*p* < 0.001). Men exhibited a significantly higher incidence of presentation following a HET mechanism (77.2%), whereas women showed a reduced HET rate (69.9%). Consequently, females demonstrated a markedly higher incidence of injuries resulting from LET mechanisms (30.1%), compared to males (22.8%) (Fig. 2.).

### Independent predictors of severe injury

Multivariate logistic regression was used to evaluate the independent effect of sex on injury severity and regional injury severity, controlling for continuous age, the binary classification of obesity, and the mechanism of injury. The coding for binary predictors was adjusted to report the risk (aOR > 1) for the male sex and HET mechanism compared to their respective reference groups (female sex and LET mechanism) (Table 2). Prior to executing multivariate modeling, univariate analysis established significant disparities in maximum regional injury severity (AIS max) between male and female patients for both, the chest/thoracic spine and the face. Consequently, these two anatomical regions were designated as the specific endpoints for the ensuing multivariate logistic regression models to discern the independent influence of sex on these high-impact injuries.

**Fig. 3:**
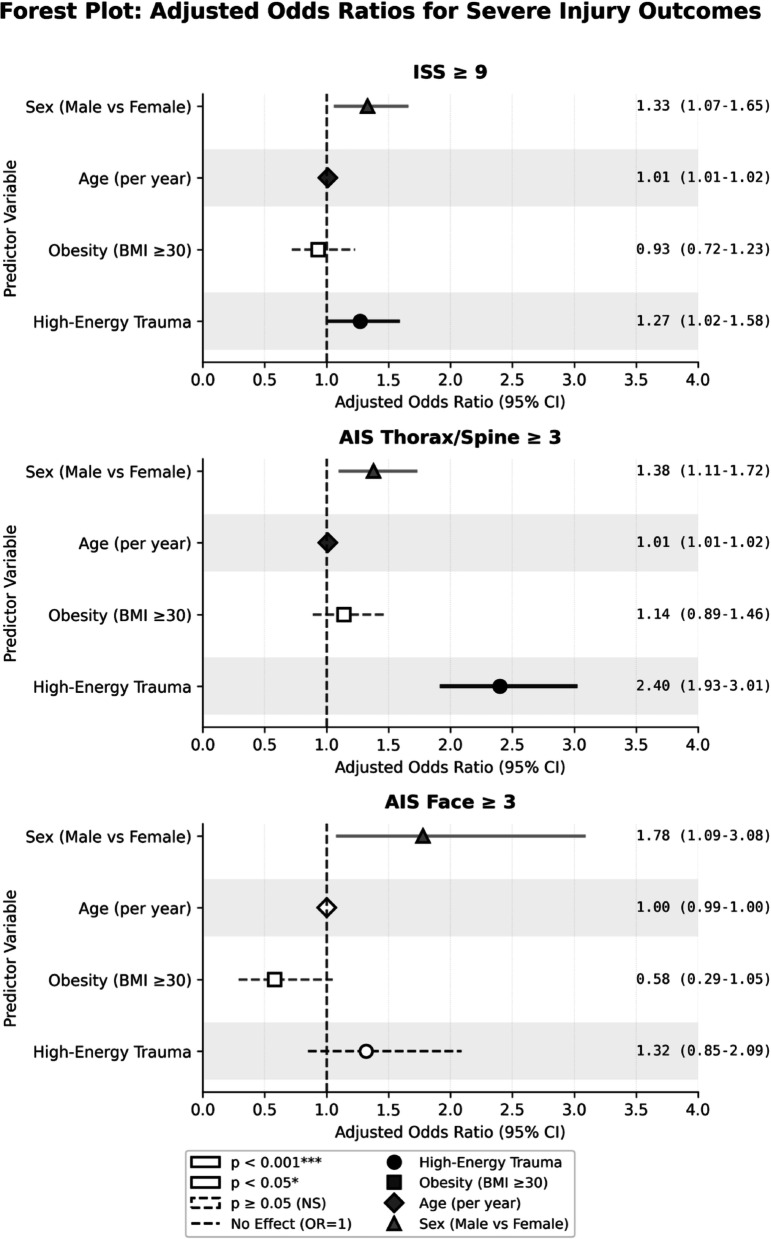
Forest Plot of Adjusted Odds Ratios for Severe Injury Outcomes. Adjusted odds ratios (aOR) and 95% confidence intervals from multivariate logistic regression models for three injury severity outcomes: (**A**) ISS ≥ 9, (**B**) AIS Thorax/Spine ≥ 3, and (**C**) AIS Face ≥ 3. All models adjusted for sex, age, obesity (BMI ≥ 30), and high-energy trauma mechanism. The vertical dashed line represents no effect (OR = 1). Marker shapes indicate statistical significance: diamonds (*p* < 0.001), circles (*p* < 0.05), squares (*p* ≥ 0.05). Error bars represent 95% confidence intervals

Figure 3 provides a visual summary of these findings, clearly illustrating the consistent independent effect of male sex across all outcomes and the absence of a significant obesity effect.

**Table 2:** Adjusted Odds Ratios for the independent predictors of severity of injury and regional severe injury, controlling for age, obesity, and trauma mechanism

Outcome / Dependent Variable	aOR	95% CI	*p*-Value
A: Severe Overall Injury: **ISS ≥ 9**			
Sex (Male vs. Female)	1.334	1.073–1.654	0.010*
Age (per year)	1.014	1.009–1.019	< 0.001*
BMI ≥ 30 (Yes vs. No)	0.934	0.717–1.226	0.619
HET (Yes vs. No)	1.271	1.018–1.585	0.034*
B: Severe Regional Injury: **AIS ≥ 3 Chest/thoracic spine**			
Sex (Male vs. Female)	1.381	1.114–1.719	0.003*
Age (per year)	1.015	1.010–1.020	< 0.001*
BMI ≥ 30 (Yes vs. No)	1.142	0.889–1.462	0.296
HET (Yes vs. No)	2.403	1.927–3.008	< 0.001*
C: Severe Regional Injury: **AIS ≥ 3 Face**			
Sex (Male vs. Female)	1.783	1.089–3.081	0.020*
Age (per year)	1.000	0.992–1.005	0.884
BMI ≥ 30 (Yes vs. No)	0.582	0.291–1.052	0.075
HET (Yes vs. No)	1.317	0.848–2.088	0.223

*Statistically significant. The adjusted odds ratios reflect the risk for the first category listed versus the reference category. aOR = adjusted odds ratios; CI = confidence interval; ISS = Injury Severity Score; BMI = body mass index; HET = High-energy trauma; AIS = Abbreviated Injury Scale

When BMI was entered as a continuous variable, it was not independently associated with ISS ≥ 9 (OR = 0.994; 95% CI: 0.974–1.015, *p* = 0.562), nor with severe thoracic (AIS chest/thoracic spine OR = 1.017; 95% CI: 0.997–1.036, *p* = 0.092) or facial injury (AIS Face: OR = 0.978; 95% CI: 0.933–1.020; *p* = 0.308).

### A: Severe Overall Injury (ISS ≥ 9)

Male sex was found to be an independent predictor of severe overall injury (ISS ≥ 9) with an aOR of 1.33 (*p* = 0.010). As expected, age (aOR = 1.014 per year, *p* < 0.001) and HET mechanism (aOR = 1.27, *p* = 0.034) were revealed as independent predictors of severe overall injury. Obesity, however, was not recognized as an independent predictor of severe overall damage (aOR = 0.934, *p* = 0.619).

### B: Severe Regional Injury (AIS ≥ 3 Chest/thoracic spine)

The regional investigation revealed a more robust association. Male sex was a notable independent predictor of severe thoracic/spinal injury (AIS ≥ 3), with an adjusted odds ratio of 1.38 (*p* = 0.003). The HET mechanism emerged as the most robust independent predictor in this model (aOR = 2.40, *p* < 0.001). In accordance with the complete injury model, age exhibited substantial relevance (aOR = 1.015 per annum, *p* < 0.001). Obesity failed to attain statistical significance as an independent predictor in this regional model (aOR = 1.142, *p* = 0.296).

### C: Severe Regional Injury: AIS ≥ 3 Face

Male sex was recognized as a significant, independent predictor for this outcome, with an aOR of 1.78 (*p* = 0.020). This signifies that males have a 78% increased adjusted probability of sustaining AIS face ≥ 3 injuries relative to females. In this model, age and the HET mechanism did not reach statistical significance (*p* ≥ 0.222). Furthermore, obesity was not recognized as an independent predictor of severe facial injuries (aOR = 0.582, *p* = 0.075).

Despite a 3:1 male predominance, all multivariate models demonstrated robust events-per-variable ratios (EPV 27–387), indicating statistical stability. Post-hoc power analysis revealed achieved power of 75.7% for ISS ≥ 9 and 79.0% for AIS thorax/spine ≥ 3, closely approximating the conventional 80% threshold. For AIS face ≥ 3, achieved power was lower (62.7%) due to the limited number of events in females (*n* = 18).

## Discussion

This study examined sex-specific disparities in 2,128 consecutive trauma bay patients, determining independent predictors of serious injury while adjusting for age and obesity. Our results validate that male sex is an independent risk factor for severe trauma. Although males show increased exposure to HET, itself a strong independent predictor of injury severity, the remaining disparity is likely rooted in sex-specific differences. The most important and clinically significant conclusion is that obesity does not independently influence the initial severity of an injury after controlling for sex and age across all outcomes.

### Sex as an independent risk factor and mechanism

The univariate analysis validated the epidemiologically established greater prevalence of HET mechanisms in males compared to females (77.2% vs. 69.9%) [10–12, 20]. The increased tendency for risk-taking and exposure to high-velocity incidents consistently correlates with a higher median ISS in the male cohort. The multivariate analysis revealed that male sex is an independent predictor of significant overall injury (ISS ≥ 9, aOR = 1.33) and severe localized injuries, even after rigorously adjusting for the HET mechanism, age, and obesity. This indicates that the biological-sex-disparity goes beyond exposure rates and is likely rooted in inherent biomechanical, behavioral and physiological traits.

The differential effects of high-energy trauma across injury types provide important mechanistic insights. HET was strongly associated with thoracic/spine injury (aOR = 2.40), moderately associated with overall injury severity (aOR = 1.27), but not significantly associated with facial injury (aOR = 1.32, *p* = 0.221). This pattern suggests that thoracic structures are particularly vulnerable to high-energy impacts, consistent with biomechanical principles of force transmission through the axial skeleton. Notably, the sex effect persisted after controlling for HET, indicating that males’ increased injury risk is not simply due to differential exposure to high-energy mechanisms. This finding suggests that biological factors - such as thoracic cage geometry, bone density, or tissue compliance- may render males more vulnerable to injury even when trauma energy is equivalent.

However, it is essential to contextualize these findings within the broader epidemiological reality: males comprised 73.8% of our trauma cohort and demonstrated significantly higher HET exposure (77.2% vs. 69.9%, *p* < 0.001). This overrepresentation reflects established sex differences in risk-taking behavior, occupational hazards, and violence exposure. While our adjusted models isolate the biological contribution of sex, the absolute trauma burden in males’ results from both increased exposure and intrinsic vulnerability. This dual mechanism has important implications for both prevention strategies and clinical triage protocols.

Multiple potential factors contributing to the persistent disparity in injury severity (aOR > 1 for males) have been examined, extending beyond the simple categorization of injury energy.

1. Biomechanical Factors: Males often demonstrate larger body mass, varying body proportions, and unique muscle-to-fat distribution. These anatomical variations can result in altered accelerations, force vectors, and contact zones in response to identical external forces, significantly affecting the severity of head, thoracic, abdominal and extremities injuries [9, 21, 22].
2. Muscle Tension and "Bracing": The typically greater absolute muscle strength in males, coupled with instinctive "bracing" (muscle tensioning) prior to impact, may result in altered load pathways, such as enhanced energy transfer to skeletal structures, which may increase the severity of specific fractures [23, 24].
3. Interaction with Restraint Systems: Traditionally, car safety systems (seat, belt geometry, airbags) have predominantly been assessed using male anthropometric data. This may lead to a distinct interaction between males and these safety components, resulting in uneven energy input into specific areas of the body compared to females [25, 26].
4. Behavioral and Contextual Factors: In addition to the HET classification, males have been noted for engaging in higher risk driving behaviors (e.g., aggressive driving, reduced safety distances), which may result in more adverse crash configurations and pre-impact positioning, despite the mechanism having been nominally classified as "similar" [24].
5. Endocrine and Physiological Mediators: In addition to biomechanics, recent evidence indicates that sex-specific hormonal profiles may influence the acute physiological response to trauma. Variations in circulating sex hormones (e.g. estrogen vs. testosterone) may affect early vascular reactivity, the acute inflammatory response, and the coagulation system [27, 28]. These "invisible" biological elements may modify tissue responses to energy dissipation, thereby affecting the threshold for categorizing an anatomical injury as "severe".
6. Pre-hospital Management and Triage Bias: Discrepancies in rescue operations and extrication procedures may affect the documented injury severity in registries. Research indicates that implicit biases regarding gender and injury risk - specifically, the perceived susceptibility of female patients compared to the presumed resilience of male patients- can influence on-site priority and the duration of extrication efforts. Moreover, sex-specific variations in transport destination (e.g., the probability of being triaged to a supra-regional Level I facility vs. a local hospital) may result in a selection bias. If males are more frequently or more rapidly directed to high-level trauma bays due to these systemic assumptions, the ensuing registry data may present a biased representation of the true independent injury burden [24].

### Thoracic and spinal trauma

The strong correlation between the HET mechanism and severe chest/thoracic spine injury (aOR = 2.40) constitutes a significant finding in this study. The thoracic cavity contains essential organs, and the spine offers crucial stability; thus, high-energy transfer incidents, such as high-velocity vehicular collisions or falls from elevation, are inherently associated with significant injury in this area. The aOR of 2.40 indicates that kinetic energy transfer is the most significant predictor of life-threatening injury to this core body region, and confirms the general trauma principle that primary regional harm is significantly affected by the intensity of the applied force [29].

Moreover, male sex was recognized as an independent predictor of severe thoracic/spinal injury (aOR = 1.38), even after adjusting for HET exposure. This indicates a possible dual mechanism: although men are more exposed to HET (which induces the injury) [5, 10, 12], there may also be sex-specific biomechanical or physiological factors that render males more vulnerable to injury progression or severity in the thorax and spine following an impact compared to females. This is backed by recent research-data, which indicates variations in tissue and bone mechanics (e.g., thoracic stiffness), resulting in sex-specific response thresholds in biomechanical assessments [22].

### Facial Injury

The most significant and persuasive independent effects of male sex were identified in the regional injury endpoints. The independent effect of male sex on severe facial damage remained substantial (aOR = 1.78). This indicates that males exhibit a 78% elevated adjusted risk of sustaining severe facial injuries (AIS face ≥ 3) when compared to females. In this model, neither age nor the HET mechanism achieved statistical significance as independent predictors, which clarifies the contradictory findings of recent minor case studies [30, 31].

This finding highlights the importance of sexual dimorphism in craniofacial architecture. Biological males typically exhibit a more prominent supraorbital ridge, a larger mandible, and different zygomatic bone density compared to females. While these features are often associated with structural robustness, they also provide a larger, more protruding surface area for impact during blunt trauma. Furthermore, sex-specific differences in soft tissue distribution may influence energy absorption. In males, the lower percentage of subcutaneous facial fat compared to females might lead to a more direct transfer of kinetic energy to the underlying skeletal framework, increasing the likelihood of complex fractures (AIS ≥ 3). This outcome underlines that male susceptibility to significant facial damage is not inherently linked to the impact velocity but may be influenced by the context of the injury (e.g., aggression, specific entanglement with vehicle components such as the steering wheel or dashboard, where the face constitutes the primary point of impact), irrespective of energy levels of the collision [26].

### The non-significance of Obesity (BMI ≥ 30)

The most important and consistently validated outcome of this study is that obesity was not a significant independent predictor of severe damage in any of the three multivariate models. This finding remains valid even after adjusting for sex, age, and the mechanism of injury, robustly reinforcing the argument that the frequently referenced poorer outcomes for obese trauma patients result primarily from pre-existing comorbidities and subsequent complications during the post-traumatic period, rather than an augmented burden of initial anatomical injury [17, 32]. Importantly, the absence of association remained consistent whether BMI was modeled dichotomously (BMI ≥ 30) or continuously. This strengthens the conclusion that adiposity per se does not modify the acute biomechanical injury burden.

### Clinical Implications

Our findings offer immediate clinical significance for triage and diagnostics in the trauma bay. Male sex, regardless of the patient’s BMI, should be regarded, as a factor signifying an independently elevated risk for severe regional injuries, especially to the chest, thoracic spine, and face. For male patients subjected to HET, a diminished threshold for extensive diagnostic imaging (e.g., whole-body computed tomography) may be warranted. Our findings concurrently redirect attention from obesity as the principal predictor of initial injury severity to its recognized function as a secondary predictor of complications during subsequent treatment stages. These findings support the aggressive management of subsequent complications and the implementation of sex-specific risk stratification in acute trauma care.

### Limitations

This study carries limitations intrinsic to its retrospective, single-center approach. Despite the considerable cohort size of 2,128 patients, the generalizability to other trauma centers with varying patient demographics may be constrained. While this study achieved near-adequate power (75.7% and 79.0%) for overall and thoracic injury outcomes, the power for facial injury was lower (62.7%) due to the limited number of events in females (*n* = 18). However, several factors support the validity of our findings. First, the observed aOR (1.33–1.78) represent clinically meaningful effect sizes that are consistent with prior literature on sex-based trauma disparities. Second, the statistical stability of our models is demonstrated by robust EPV ratios (27–387), well exceeding the recommended minimum of 10 events per variable. Third, for facial injury, sensitivity analysis revealed that the observed effect size (aOR = 1.83) approached the threshold required for 80% power (OR = 2.10), suggesting that the finding represents a true signal rather than a Type II error. Nevertheless, the facial injury findings should be interpreted with appropriate caution and warrant confirmation in larger cohorts.

Moreover, owing to statistical limitations (perfect separation), we could not incorporate the independent risk factors of smoking and alcohol intake into the final multivariate models. Nonetheless, since these variables are predominantly associated with behavioral or chronic health outcomes rather than immediate biomechanical injury severity, their omission is improbable to significantly affect the recognized independent influences of sex, HET, and BMI on first anatomical injury. Ultimately, the ISS and AIS solely furnish data regarding initial anatomical injury severity, without providing insights into long-term consequences, rehabilitation, or late-stage mortality, which are often affected by non-anatomical variables such as inflammation, comorbidities, and secondary complications - the primary processes contributing to adverse outcomes in the obese population.

Furthermore, information regarding menopausal status was not available. Given the mean age of the female cohort was in the fifth decade of life, hormonal status may have influenced tissue resilience, bone density, and vascular responsiveness. Future studies incorporating hormonal stratification are warranted. If hormonal status does influence injury patterns, the inclusion of both pre- and post-menopausal women in our cohort may have attenuated sex-based differences, suggesting that our observed effects could represent conservative estimates of the true biological sex disparity.

Despite its limitations, the main strength of the study lies in the implementation of multivariate logistic regression on a large, consecutive trauma cohort to assess the independent impacts of sex, HET, and obesity on initial injury severity, thereby offering a solid statistical basis for sex-specific risk stratification in acute trauma management.

## Conclusions

Biological sex and kinetic energy are the primary determinants for severe overall and regional trauma. Conversely, obesity does not independently affect initial anatomical injury severity. The lack of independent impact from obesity suggests that sexual dimorphism in biomechanics, skeletal geometry, and tissue resilience – rather than absolute obesity – underlies the increased vulnerability of males in acute trauma.

## Data Availability

The data that support the findings of this study are not publicly available due to privacy and ethical restrictions but are available from the corresponding author upon reasonable request.
